# 513. Evaluating the safety of baricitinib versus tocilizumab in hospitalized patients with COVID-19

**DOI:** 10.1093/ofid/ofad500.582

**Published:** 2023-11-27

**Authors:** Kendall Brickel, Lyndsi Meyenburg, Dusten T T Rose, Neil Pan

**Affiliations:** Dell Seton Medical Center at the University of Texas , Austin, Texas; Ascension Seton, Austin, Texas; Dell Seton Medical Center at the University of Texas, Austin, Texas; Ascension Seton, Austin, Texas

## Abstract

**Background:**

Tocilizumab and baricitinib are immunomodulators that combat immune dysregulation observed in patients with COVID-19. To date, no studies have explicitly looked at the safety of baricitinib versus tocilizumab in patients with COVID-19.

**Methods:**

This evaluation is a multicenter, retrospective cohort study of adult patients treated with either baricitinib or tocilizumab for COVID-19 within the Ascension Seton network from May 1st, 2020 - August 31st, 2022. A control group consisted of patients who would otherwise have qualified for treatment but did not receive either agent. The primary objective was the incidence of adverse effects in patients with COVID-19 treated with baricitinib versus tocilizumab. Secondary objectives include rates of thromboembolism, hematologic toxicity, hospital-acquired infection, hepatic enzyme elevation, gastrointestinal perforation, death, and any adverse effect believed to be secondary to or that resulted in the discontinuation of either study medication. A chi-squared test was used to assess the primary outcome. Secondary outcomes were analyzed using appropriate statistical tests.

**Results:**

Of the 737 patient encounters screened, 145 patients were included in each group. Adverse event rates were 112/145 (77.2%) for the baricitinib group and 114/145 (78.6%) for the tocilizumab group (p = 0.78). In the control group, adverse event rates were 86/145 (59.3%), and a Kruskal–Wallis test revealed a difference between the three groups (p = 0.007). Twenty-two (15.3%) of patients who received baricitinib experienced a documented adverse drug reaction based on progress notes from the provider versus one (1.4%) patient in the tocilizumab group (p < 0.001). Baricitinib was associated with significantly higher rates of thrombocytosis (p = 0.008).

**Figure:**
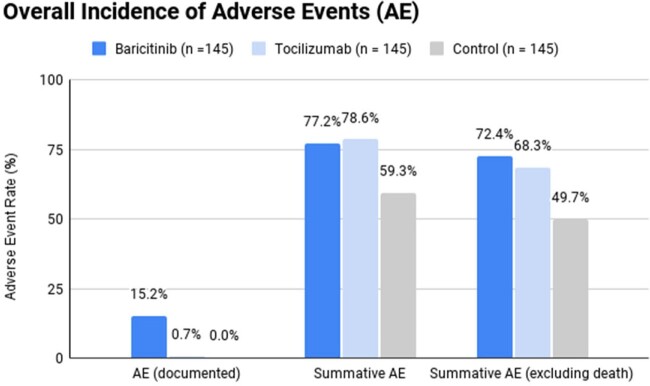

**Conclusion:**

Among our study population, baricitinib was associated with increased rates of adverse events documented in progress notes. However, baricitinib was comparable to tocilizumab regarding adverse events, including hepatotoxicity, hematologic toxicities, thrombotic risk, gastrointestinal perforation, hospital-acquired infections, and death. Overall, baricitinib was shown to be a safe alternative to tocilizumab, and patients receiving either agent are at increased risk for adverse events.

**Disclosures:**

**All Authors**: No reported disclosures

